# Polystyrene-Templated Microstructure Engineering of Aerosol-Deposited WO_3-x_ Films for Enhanced Hydrogen Sensing

**DOI:** 10.3390/ma19143079

**Published:** 2026-07-17

**Authors:** Xin Zhang, Yuan-Bo Zhang, Jong-Min Oh, Jie Wei

**Affiliations:** 1 Suzhou Institute of Biomedical Engineering and Technology, Chinese Academy of Sciences, Suzhou 215163, China; zhangx@sibet.ac.cn; 2 Department of Electronic Materials Engineering, Kwangwoon University, Seoul 01897, Republic of Korea; 3 School of Electronic and Information Engineering, Suzhou Polytechnic University, Suzhou 215104, China; 4 Suzhou Sanse Sensing Technology Co., Ltd., Suzhou 215000, China

**Keywords:** powder aerosol deposition, hydrogen sensor, non-stoichiometric tungsten oxide, polystyrene sacrificial template, lithium-ion battery safety monitoring

## Abstract

High-performance hydrogen sensors are crucial for the safe operation of lithium-ion batteries with regard to thermal runaway monitoring, which has driven extensive research. In this work, in order to satisfy the urgent requirement of H_2_ sensors for lithium-battery safety monitoring, a polystyrene sacrificial phase was introduced to fabricate porous WO_3_-based sensing films with tunable oxygen stoichiometry. Through PAD (Powder Aerosol Deposition) followed by sintering in air at 500 °C, the pore structure and defect chemistry of WO_3-x_ films were simultaneously regulated. Structural characterizations show that polystyrene can be completely removed during sintering without changing the main γ-WO_3_ phase, while interconnected pores are generated and the films evolve from near-stoichiometric WO_3_ toward oxygen-deficient WO_3-x_ with increased oxygen-vacancy concentration. Meanwhile, the PAD-derived amorphous matrix encapsulated nanocrystalline is largely preserved, providing stable charge-transport pathways. The porous structure improves hydrogen diffusion and reaction accessibility, while bulk reduction and oxygen-vacancy enrichment enhance surface reactivity and charge transfer, together leading to improved H_2_ sensing performance. Among all samples, WP5 exhibits the best overall performance, achieving stable H_2_ detection over a wide range of 10 ppb to 20,000 ppm at 120 °C, together with extraordinary response. These results demonstrate that this strategy is an effective route for simultaneously engineering porosity and defect chemistry in WO_3-x_-based sensing films, and highlight the potential of Pd/PS–WO_3-x_ sensors for lithium-ion battery thermal runaway warning.

## 1. Introduction

Lithium-ion batteries (LIBs) underpin electrified transportation and grid storage, yet thermal runaway (TR) remains a critical safety concern. Conventional battery management strategies largely rely on electrical/thermal observables (e.g., voltage, current, temperature) that often change conspicuously only after internal reactions have accelerated, leaving limited lead time for mitigation [1,2,3]. In contrast, gas evolution provides a chemically direct window into early abuse and degradation processes, and gas sensing has therefore emerged as a promising route for early TR warning. Recent reviews and experimental studies highlight that hazardous gases (CO, CH_4_, C_2_H_4_, H_2_, electrolyte vapors, etc.) can be detected before macroscopic failure signatures become apparent, enabling earlier risk estimation and intervention [4]. Among TR-related gases, hydrogen (H_2_) is particularly attractive as an early indicator because it can appear at trace levels during pre-runaway venting and electrolyte/electrode side reactions (SEI decomposition, reactions of lithium with water, binder pyrolysis, recombination of atomic hydrogen). Importantly, selective trace H_2_ detection has been demonstrated to provide reliable early warning across multiple LIB formats and abuse conditions, underscoring the practical value of hydrogen sensing for battery safety monitoring [5].

Metal-oxide semiconductors (MOSs) like SnO_2_, ZnO, and In_2_O_3_ are widely investigated for resistive gas sensing due to their robustness and manufacturability [6,7,8,9], but their sensing behavior is mainly governed by surface adsorption/reaction processes and may suffer from high operating temperature, limited selectivity, or environmental interference. However, tungsten oxide (WO_3_) is a particularly compelling H_2_-responsive material because its interaction with hydrogen is not limited to surface oxygen ionosorption and charge transfer [10,11,12,13]. WO_3_ also exhibits gasochromic properties, implying a coupled surface–bulk reaction pathway that can amplify the detectable signal [14,15,16].

From a manufacturing perspective, there is strong motivation to develop sensor films that are not only sensitive but also scalable. Prior studies have demonstrated the unique advantages of powder aerosol deposition (PAD) for fabricating WO_3_-based hydrogen sensors, in particular its capability to produce an amorphous matrix encapsulated nanocrystals architecture [17,18]. To further realize the coordinated optimization of the WO_3_ microstructure and bulk defects while preserving this PAD-enabled amorphous matrix encapsulated nanocrystalline structure, introducing organic templates for structural design is a common and effective strategy [19]. Among various organic templates, polystyrene (PS) offers merits including wide availability, low cost, facile dry mixing with inorganic powders, and controllable particle size and morphology. During thermal treatment, PS can undergo pyrolysis and generate gaseous products such as CO_2_: on the one hand, it acts as a sacrificial space-holding phase that leaves behind porous structures after sintering [20], and on the other hand, the local pyrolysis atmosphere may create a mildly reducing environment, which is expected to enable simultaneous regulation of the bulk stoichiometry of WO_3_ and the concentration of oxygen vacancies. Consequently, PS is frequently used as a sacrificial template in porous ceramics, catalytic materials, and gas-sensing materials, showing distinctive advantages in increasing specific surface area, optimizing gas-diffusion pathways, and tuning the reduction degree [21].

Based on these considerations, this work attempts to combine the PS sacrificial-templating strategy with the PAD process. PS–WO_3_ composite mixed starting powders were prepared via simple mechanical mixing, enabling the co-deposition of PS and WO_3_ under identical processing conditions to construct PS–WO_3_ composite films. Subsequently, PS was removed by high-temperature calcination in synthetic air, and the effects of PS content in the precursor powders on the film porosity, crystalline phase composition, bulk reduction degree, and oxygen-vacancy distribution were systematically investigated, as illustrated in Figure S1. This work presents a comprehensive study from the perspectives of structural evolution, defect regulation, and hydrogen-sensing performance, elucidating the synergistic roles of PS as a sacrificial phase in bulk reduction and porous microstructure design in PAD-deposited WO_3_, and discussing its potential for wide-range, high-sensitivity hydrogen sensors for lithium-battery safety applications.

## 2. Materials and Methods

### 2.1. Sample Preparation

PS powder (Avention Co., Ltd., Icheon-si, Republic of Korea; AR; ~2 μm microsphere diameter) was employed as a sacrificial template to engineer the microstructure of PAD-deposited WO_3_-based films. PS was dry-mixed with the WO_3_ starting powder (Avention Co., Ltd., Icheon-si, Republic of Korea; AR; 0.5~5 μm particle size) to form PS–WO_3_ composite starting powders with PS volume fractions of 3, 5, 10, and 20 vol%. PS–WO_3_ composite films were fabricated using powder aerosol deposition. Helium was selected as the carrier gas with a flow rate regulated by a mass flow controller fixed at 3 L·min^−1^. During deposition, the substrate was reciprocally scanned beneath the nozzle for two passes to ensure uniform film formation. Optical-grade quartz plates were used as substrates and were pretreated by ultrasonic cleaning followed by vacuum drying to remove surface contaminants and adsorbed moisture, thereby improving film–substrate adhesion and reproducibility. The detailed PAD parameters are summarized in Table S1. With this process, PS–WO_3_ films with sufficient thickness, compactness, and adhesion could be rapidly prepared at room temperature, providing reliable templates for subsequent microstructure/defect regulation studies. According to the PS content introduced in the starting powders, the samples were denoted as WP3, WP5, WP10, and WP20, respectively. After PAD of the PS–WO_3_ composite films, the samples were thermally treated in air to remove the PS sacrificial phase and stabilize the WO_3_ framework. Specifically, the films were heated to 500 °C at a ramp rate of 2 °C·min^−1^ and held for 2 h, followed by furnace cooling to room temperature. The annealing temperature of 500 °C was selected to ensure the oxidative decomposition and removal of the PS sacrificial phase in air. This temperature is sufficiently high to eliminate PS-related organic residues, as confirmed by the disappearance of the characteristic PS Raman bands after calcination, while avoiding excessive thermal coarsening or severe structural degradation of the PAD-derived WO_3_ framework. The detailed PS contents and corresponding sample labels are summarized in Table S2 in the Supplementary Materials. After thermal treatment and removal of the PS sacrificial phase, a Pd catalytic overlayer with a nominal thickness of approximately 3 nm was deposited onto all WO_3-x_ films by magnetron sputtering. The Pd layer was introduced to promote H_2_ dissociation and hydrogen spillover during gas-sensing measurements. Unless otherwise specified, the hydrogen-sensing tests described below were performed using Pd/WO_3-x_ sensor structures rather than pristine WO_3-x_ films.

### 2.2. Structural Characterization

To verify PS removal by calcination, Raman spectroscopy was used to track PS signature bands in the as-deposited composite films and after thermal treatment. The evolution of film morphology and microstructure induced by PS templating was examined by SEM, with particular attention to surface roughness changes and the possible emergence of pores/voids after calcination. Film thickness variations as a function of PS fraction were quantified by stylus profilometry. XRD and XPS were further employed to evaluate whether PS-assisted thermal processing altered the crystalline phase and bulk reduction state of WO_3-x_, respectively.

The PS used in this work was commercially available polystyrene microspheres, and its structure and morphology were systematically characterized, as shown in Figure S2. The XRD pattern of the PS sample (Figure S2a) exhibits no sharp Bragg diffraction peaks, but instead shows a broad and diffuse scattering band, indicating the absence of long-range crystalline order and suggesting that the polymer chains possess only short-range packing correlations. For amorphous polymers such as PS, this diffuse scattering is generally associated with the average interchain spacing and the short-range ordering arising from the packing of aromatic rings and chain segments. Its broad profile and relatively weak intensity are consistent with the low overall scattering cross-section and intrinsically amorphous nature of polymeric materials. In contrast, the Raman spectrum (Figure S2b) displays the typical fingerprint features of an aromatic polymer. In the low-wavenumber region, several bands related to the benzene-ring framework can be identified, among which the band at ~620 cm^−1^ is assigned to ring deformation/skeletal bending vibrations, while signals in the ~750–800 cm^−1^ range are associated with out-of-plane C–H bending of the aromatic ring. Most notably, a strong and sharp peak appears at ~1001 cm^−1^, which is generally attributed to the breathing mode of the aromatic ring and is widely recognized as the most characteristic Raman fingerprint of PS. A further band near ~1030 cm^−1^ can be assigned to in-plane C–H deformation and skeletal ring vibrations. In addition, the weak features observed in the 300–500 cm^−1^ region are likely related to low-frequency ring deformation/torsional modes coupled with main-chain vibrations, whereas the pronounced rising background below 200 cm^−1^ is typically caused by edge noise in the low-wavenumber region. Taken together, the combination of these characteristic peak positions and spectral features confirms that the sample is polystyrene. These features can also serve as diagnostic markers for the subsequent template removal process in PS–WO_3_ composite films. When the characteristic aromatic-ring bands near ~1000 cm^−1^ (and ~1030 cm^−1^) are significantly weakened or disappear after heat treatment, the PS template can be regarded as effectively removed; if these peaks remain, incomplete removal of the organic template or the presence of carbonaceous residues should be suspected. The SEM image, shown in Figure S3, reveals that the PS powder consists of near-spherical particles with a micrometer-scale morphology and an average particle size of approximately 4.4 μm.

### 2.3. Gas Sensing Measurement

The H_2_ sensing performance of the Pd/WO_3-x_ devices were measured on a self-built four-channel dynamic gas sensor measurement system integrating a gas-delivery manifold, a programmable multistage dilution module f, a sealed measurement chamber, and a data-acquisition unit. The setup enables parallel evaluation of up to four sensors at a sampling rate up to 10 Hz. Target-gas concentrations were produced by diluting certified H_2_ mixtures with synthetic air (79 vol% N_2_ and 21 vol% O_2_), providing a maximum dilution factor of 2000× (e.g., 2 vol% H_2_/air to 10 ppm, or a 100 ppm source to 50 ppb). The test chamber includes an integrated heater capable of reaching 300 °C and a quartz window for in situ spectroscopic monitoring during gas exposure.

The H_2_-sensing performances of Pd/WO_3-x_ films prepared with different PS fractions were measured under the same testing protocol as the reference sensor. The sensor response was defined as S = Rair/Rgas, and the response/recovery times were extracted as the time required to reach 90% of the total resistance change upon switching between synthetic air and the target gas. Cross-interference tests against typical co-existing gases (e.g., CO, H_2_S, NH_3_, NO_2_, and NO) were performed under identical conditions to assess the selectivity impact of PS-enabled microstructure tuning.

## 3. Results and Discussion

### 3.1. Structural and Morphological Characterization

**Figure 1:**
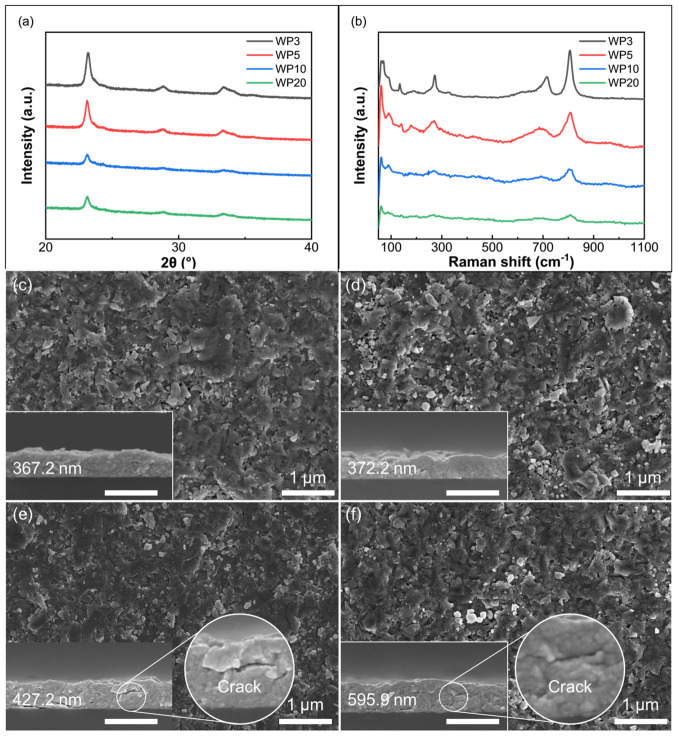
Structure characterization of as-prepared samples. (**a**) XRD; (**b**) Raman spectrum and SEM images of (**c**) WP3; (**d**) WP5; (**e**) WP10; (**f**) WP20.

To verify whether the PS sacrificial template introduced during fabrication could be completely removed during the subsequent sintering process, and to examine the influence of thermal decomposition during sintering on the crystal structure of WO_3_, XRD measurements were first performed on the sintered samples containing different amounts of PS. As shown in Figure 1a, no broad diffuse amorphous scattering peak attributable to PS was observed in any of the diffraction patterns, and all detectable reflections could be indexed exclusively to WO_3_, indicating that polystyrene had been essentially fully decomposed and volatilized under air at 500 °C, leaving no ordered or disordered organic residue in the final films. Further comparison reveals that the diffraction peak positions of all samples agree well with the standard PDF# No. 97-001-7003 and can be assigned to monoclinic γ-WO_3_, demonstrating that the introduction of PS and its subsequent thermal decomposition did not alter the primary crystal phase of WO_3_. Compared with the starting powder, the film samples retained a relatively strong diffraction peak near 2θ ≈ 23.147°, corresponding to the (002) plane, whereas the reflections from other crystallographic planes were markedly weakened or nearly absent, indicating a pronounced preferred orientation. This behavior is closely associated with the PAD process, in which high-energy particles impact the substrate along the normal direction and undergo selective lattice “survival”. In γ-WO_3_, the (002) plane is parallel to the c-axis and can be regarded as a W–O layered plane (d ≈ 0.37 nm), with adjacent layers connected by relatively weak van der Waals interactions. Under high-strain-rate impact, this structure is more prone to interlayer sliding for energy dissipation rather than complete fragmentation, thereby favoring its retention during deposition and ultimately resulting in a strongly (002)-oriented film structure [22].

Building on the XRD identification of phase composition and preferred orientation, Raman spectroscopy was further employed to probe, from the perspective of local bonding structure, the effects of PS removal by thermal decomposition and sintering on the bond length, bond angle, and defect state of WO_3_. The results are presented in Figure 1b. First, no characteristic vibrational features associated with PS were observed in the Raman spectra of any sample. In particular, the strong and sharp peak near 1001 cm^−1^, which is assigned to the “breathing” mode of the benzene ring and is regarded as the most representative Raman fingerprint of PS, was absent. Meanwhile, the characteristic band around 620 cm^−1^, typically attributed to skeletal benzene ring bending/deformation vibrations, also completely disappeared. The absence of these two characteristic PS-related bands is consistent with the lack of PS-derived amorphous scattering in the XRD patterns, further confirming that the PS was fully pyrolyzed and removed from the films during sintering, and therefore did not interfere with the final Raman response of WO_3_.

For near-stoichiometric monoclinic WO_3-x_, the vibrational mode of the lattice at low frequency (~135 cm^−1^) is a characteristic fingerprint of this phase, reflecting its low-symmetry framework structure. The band near 270 cm^−1^ is assigned to the O–W–O bending vibration, while the peaks at approximately 714 and 808 cm^−1^ correspond to the stretching vibrations of bridging O–W–O and terminal W–O bonds, respectively. With increasing PS content in the starting powder, the Raman features of the sintered samples exhibited a systematic evolution. Specifically, the low-frequency fingerprint peak at 135 cm^−1^ gradually weakened and nearly disappeared in the sample containing 20 vol% PS, indicating that the overall structural order of the WO_3_ framework and the characteristic monoclinic distortion were partially suppressed, while lattice disorder and local structural distortion became significantly enhanced. This observation is consistent with the more severe bulk reduction and possible partial amorphization under high-PS conditions. Meanwhile, the intensities of the W–O–W and terminal W–O stretching bands at 714 and 808 cm^−1^ decreased overall, and the full width at half maximum of the 714 cm^−1^ peak broadened markedly, suggesting a wider distribution of W–O bond lengths and O–W–O bond angles, as well as a more heterogeneous local coordination environment. Such changes are commonly associated with the introduction of oxygen vacancies and the increased distortion of WO_6_ octahedra. In the WP5 sample, a new and relatively strong band emerged at ~950 cm^−1^ [23,24]. This high-frequency vibration is generally associated with highly shortened terminal W=O bonds or local vibrational modes related to W^5+^ species in tungsten bronze/non-stoichiometric WO_3-x_, indicating that the reducing atmosphere generated during PS pyrolysis significantly increased the reduction degree of WO_3_ not only at the surface but also in the bulk, thereby inducing the formation of more defect structures containing short W=O bonds and highly distorted WO_6_ octahedra [24]. Taken together, the XRD and Raman results suggest that, while the PS sacrificial phase was effectively removed, its pyrolysis-induced reduction effect further modulated the bulk redox state and local structure of the WO_3_ films, thereby providing a structural basis for the subsequent enhancement of the hydrogen-sensing response.

In terms of morphology, the SEM characterization results of samples with different PS contents are shown in Figure 1c–f, with the insets presenting the cross-sectional morphology and corresponding film thickness information. It can be seen that the WO_3_ films prepared by the PAD process and subsequently sintered exhibit uniform macroscopic coverage and continuous film formation, while their surfaces display a relatively rough granular texture. This feature is closely associated with the “hammering–fracturing–rearrangement” behavior of aerosol particles during high-velocity impact consolidation at room temperature. The cross-sectional images further show that, under identical processing parameters, the film thickness falls within the submicrometer-to-micrometer range, and the interface between the film and the quartz substrate is compact and well bonded, with no obvious delamination observed, demonstrating that the PAD process can achieve strong film–substrate adhesion under ambient conditions. With the introduction and gradual increase in PS in the starting powder, local micropores and microcracks with characteristic sizes in the order of hundreds of nanometers become clearly visible both on the film surface and within the cross-section after sintering, indicating that PS was effectively co-deposited as a space-occupying phase during deposition and was subsequently completely removed through pyrolysis and oxidation during heat treatment, thereby leaving voids at its original locations [25]. Notably, under the same carrier gas flow rate and scanning parameters, the cross-sectional SEM results reveal that the film thickness increases significantly with increasing PS content. This can be attributed to the additional buffering and damping effect introduced by the polymer–ceramic mixed powder during PAD, which alleviates excessive erosion of the precursor layer and secondary sputtering caused by inorganic particles, thereby improving the effective deposition efficiency [25]. Meanwhile, after PS removal during sintering, more abundant pores and interconnected channels are formed throughout both the film interior and surface. The simultaneous increase in film thickness and pore population expands the effective reaction volume and the three-dimensional scale of gas diffusion pathways, reflecting a progressive development of the porous architecture with increasing PS content. However, when the PS volume fraction exceeds approximately 10%, the excessively high organic content within the film leads to rapid gas release from the interior during sintering at 500 °C. Because the resulting local stress cannot be dissipated in time, some samples develop horizontally extended cracks within the WO_3_ layer, as highlighted in the enlarged images in Figure 1e,f.

It should be noted that the active sensing layers are substrate-supported thin films with very small mass; conventional BET measurements are not suitable for accurately quantifying their surface area or pore-size distribution. Therefore, in this work, the pore evolution was evaluated mainly from SEM and cross-sectional SEM observations. The enhanced sensing performance is discussed as a coupled result of pore formation, modified gas diffusion, oxygen-vacancy enrichment, and preserved charge-transport pathways, rather than being attributed solely to an increase in specific surface area.

Because the sensing activity of gas-sensitive materials is closely related to their surface chemical composition and elemental valence states, it is necessary to systematically analyze the chemical states of the WO_3-x_ films sintered from samples with different PS contents. XPS measurements were therefore carried out on all sintered samples, and the results are shown in Figure S4. The XPS survey spectra indicate that, apart from the unavoidable signal from adventitious surface carbon, only the characteristic peaks of tungsten and oxygen were detected in all samples, with no evidence of other impurity elements. This confirms that, after complete removal of the PS sacrificial phase, the films remained chemically composed of WO_3_ without introducing any additional interfering species. The survey spectra of samples with different PS contents exhibit essentially identical peak positions, indicating that the elemental constituents of the films did not change with PS addition; however, variations in peak intensity and relative peak area suggest systematic differences in their internal redox state and defect concentration.

**Figure 2:**
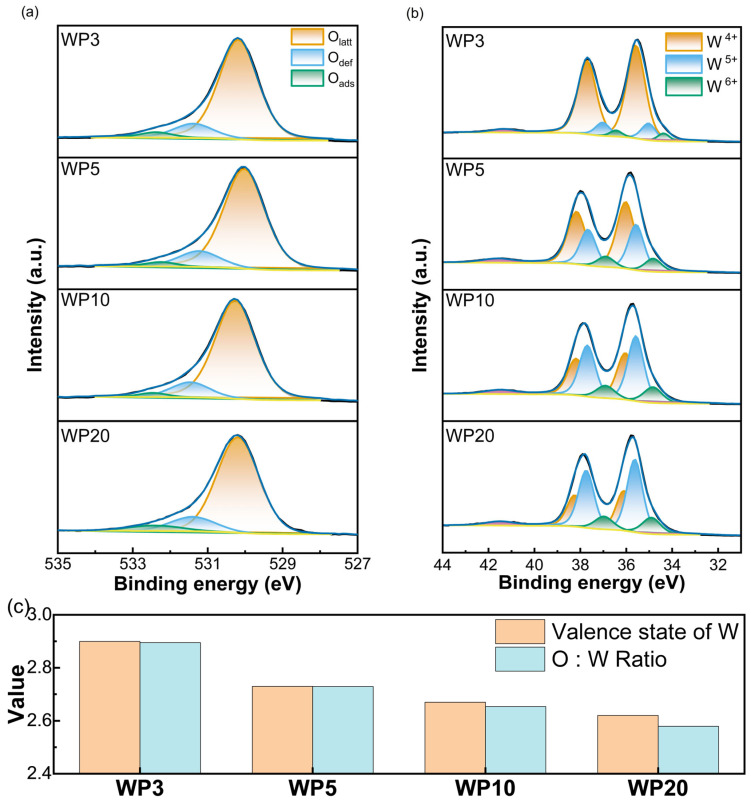
Chemical states of samples. (**a**) O 1s; (**b**) W 4f; (**c**) calculation results.

To further quantify the influence of PS content on the redox state of WO_3_ and the concentration of oxygen vacancies, high-resolution spectra of the W 4f and O 1s regions were collected and deconvoluted, as shown in Figure 2. These results reveal the evolution of the bulk reduction degree and tungsten valence-state distribution with increasing PS content. Figure 2a presents the high-resolution W 4f spectra and their fitting results. The characteristic spin–orbit doublet of W 4f7/2 and W 4f5/2 can be deconvoluted into multiple components assigned to W^6+^ and lower-valence tungsten species (W^5+^/W^4+^) [26,27]. The relative area ratios of these fitted components can therefore be used to evaluate the reduction degree of WO_3_ and the evolution of tungsten valence-state distribution with varying PS content. Figure 2b shows the deconvoluted high-resolution O 1s spectra. The O 1s signal can be resolved into three components, namely lattice oxygen (O_latt_), defect-related oxygen (O_def_), and surface chemisorbed oxygen (O_ads_). Among them, O_latt_ corresponds to O^2−^ in the WO_3_ lattice, O_def_ reflects oxygen-deficient environments associated with oxygen vacancies and non-stoichiometric WO_3-x_, and O_ads_ is related to surface-adsorbed species such as hydroxyl groups and carbonates. The XPS-derived O/W ratios of WP3, WP5, WP10, and WP20 are approximately 2.90, 2.73, 2.65, and 2.58, respectively, corresponding to an increasing degree of oxygen deficiency with increasing PS content. However, the best sensing performance is obtained by WP5, indicating that an intermediate oxygen-deficiency level is more favorable than excessive reduction. By comparing the relative area fractions of these components in samples with different PS contents, the combined regulatory effect of PS-sacrificial-phase pyrolysis on bulk reduction, oxygen-vacancy generation, and surface adsorbates in WO_3_ can be elucidated from the perspective of chemical states, thereby providing an important basis for understanding the subsequent differences in hydrogen-sensing performance.

Based on the qualitative analysis, semi-quantitative fitting of the high-resolution XPS spectra was further performed for different samples to clarify the regulatory effect of PS-sacrificial-phase pyrolysis on the bulk oxygen content and tungsten valence state of WO_3_. The O/W atomic ratio, calculated from the area ratio between the lattice-oxygen component and the W-related spectral peaks, shows that the WP3 sample has an O/W value of approximately 2.90, which is very close to the theoretical stoichiometric value of 3 for WO_3_. This indicates that, when the PS volume fraction is only 3%, the sintered film remains nearly stoichiometric overall, in good agreement with the XRD and Raman results. As the PS content in the starting powder increases from 5% to 10% and 20%, the O/W ratio decreases progressively to 2.73, 2.65, and 2.58, respectively, indicating a gradual depletion of lattice oxygen, a continuous increase in the non-stoichiometric WO_3-x_ component, and a monotonic rise in oxygen-vacancy concentration with increasing PS content. This trend is consistent with the mechanism in which PS undergoes pyrolysis during heat treatment to generate gaseous products such as CO_2_, thereby creating a locally reducing atmosphere that promotes oxygen loss from bulk WO_3_. These results suggest that PS acts not only as a geometric sacrificial template for pore formation, but also as an effective bulk reducing agent within the film. In parallel, peak deconvolution of the high-resolution W 4f spectra was used to extract the area fractions of tungsten species with different valence states, from which an apparent average valence-state descriptor was established. As concluded in Figure 2c, the results show that this parameter is about 2.90 for WP3 and then decreases successively to 2.73 for WP5, 2.67 for WP10, and 2.62 for WP20, indicating a progressive decrease in the fraction of W^6+^ accompanied by a continuous increase in the proportion of W^5+^/W4+ species. Combined with the systematic decline in the O/W atomic ratio, it can be concluded that a higher PS content leads to a stronger reducing effect of pyrolysis-generated gases within the film, which facilitates the migration of lattice oxygen and the reduction in W centers, thereby driving the evolution of WO_3_ toward WO_3-x_ on the bulk scale. Overall, the quantitative XPS results clearly verify the chain process of “PS pyrolysis → local reducing atmosphere → bulk oxygen loss → reduction in W valence state”, providing solid chemical evidence for the role of oxygen vacancies and bulk reduction in the enhanced hydrogen-sensing performance discussed later.

### 3.2. Sensing Performance

The operating temperature of metal-oxide gas sensors not only directly governs charge-carrier transport and the kinetics of surface adsorption–reaction–desorption processes, but also serves as an important tuning parameter for adapting to variations in ambient temperature and humidity and buffering external operating fluctuations in practical lithium-ion battery applications. For hydrogen sensors intended for the early warning of thermal runaway, it is necessary to achieve sufficiently high response and fast kinetics at a moderate operating temperature, while also avoiding the increased power consumption and accelerated device aging associated with excessively high temperatures. Therefore, it is essential to systematically investigate the temperature-dependent behavior of samples with different PS contents over a certain temperature range in order to identify an optimized operating window that balances sensitivity and practical applicability. Based on these considerations, the temperature-dependent hydrogen-sensing performances of four samples with different PS contents (WP3, WP5, WP10, and WP20) were evaluated under identical catalytic conditions in this section. A Pd catalytic layer with a nominal thickness of approximately 3 nm was uniformly deposited on the surface of all samples by magnetron sputtering. The operating temperature was then varied from 40 to 200 °C, and the dynamic response curves of the devices toward 2 vol% H_2_ in air were recorded at each temperature point, as shown in Figure S5.

**Figure 3:**
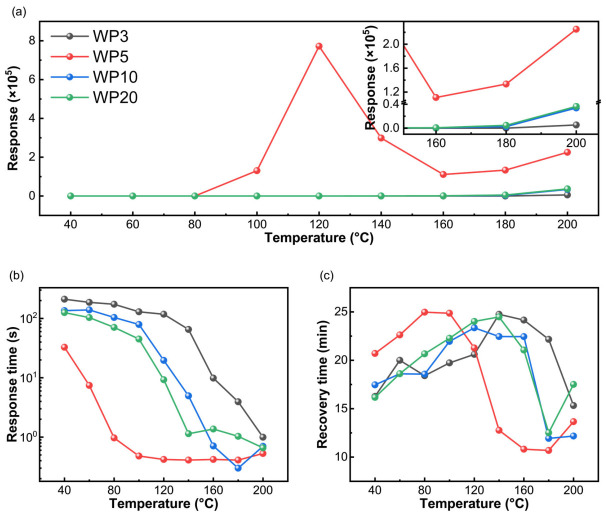
Optimization of the operating temperature: (**a**) sensing response, (**b**) response time, and (**c**) recovery time of the prepared sensors.

By comparing the response magnitude as well as the response/recovery characteristics of different samples at each temperature, the effects of PS-sacrificial-phase-induced bulk reduction and pore-structure evolution on the optimal operating temperature and high-concentration hydrogen-sensing behavior can be systematically analyzed. The steady-state response values calculated from the dynamic response curves are presented in Figure 3. It can be seen that the Pd/PS–WO_3-x_ devices treated with the PS sacrificial phase also exhibit a typical volcano-shaped temperature dependence over the range of 40–200 °C. In the low-temperature region, the response increases markedly with increasing operating temperature, reaches a maximum at around 120 °C (approximately 8.1 × 10^5^), and then gradually decreases upon further heating. Correspondingly, both the response and recovery times become shorter overall as the temperature rises, and a favorable compromise between high response magnitude and rapid kinetics is achieved near 120 °C, indicating that the adsorption/reaction and desorption processes are in a relatively optimal dynamic balance at this temperature. The decrease in the optimal operating temperature is likely associated with the combined effects of PS-induced oxygen-vacancy enrichment and porous-structure formation. Oxygen vacancies can promote oxygen activation and charge transfer, whereas the porous architecture can improve gas accessibility and reduce diffusion limitations. However, the present data do not allow a complete quantitative separation of these two contributions. Further impedance spectroscopy and activation-energy analysis will be required to determine the relative importance of defect-mediated surface reactivity and diffusion enhancement.

It should be noted that the optimum operating temperature of the samples in this work is clearly lower than that of the WO_3_ films directly prepared by PAD in previous research [18] (≈160 °C). This difference can be attributed to the bulk reduction and porous-structure construction induced by PS sacrificial-phase pyrolysis. On the one hand, PS pyrolysis creates a locally reducing environment within the film, significantly increasing the concentration of oxygen vacancies and the proportion of W^5+^ in WO_3-x_, thereby lowering the activation energy for the reaction between surface-adsorbed oxygen species and hydrogen and enabling sufficiently rapid surface reactions at lower temperatures. On the other hand, the interconnected channels and rough porous surface formed after PS removal shorten the diffusion path of hydrogen within the film and alleviate mass-transfer limitations, thus shifting the response maximum toward lower temperatures. Therefore, the synergistic effects of PS-sacrificial-phase-induced bulk reduction and pore-structure optimization allow the Pd/PS–WO_3-x_ devices to achieve hydrogen-sensing performance at around 120 °C that is comparable to, or even better than, that of untreated WO_3_ at higher temperatures, making them more suitable for the practical requirements of lithium-ion battery safety monitoring in terms of moderate operating temperature and reduced power consumption.

Taken together, the quantitative XPS results, SEM morphology, and temperature-dependent sensing behavior indicate that the superior hydrogen response of WP5 originates from an optimal balance among the degree of bulk reduction, the integrity of the pore structure, and the semiconductor transport characteristics at this composition. On the one hand, when the PS content increases from 3% to 5%, the reductive species released during PS pyrolysis lower the O/W ratio from 2.90 to 2.73, indicating that WP5 contains significantly more oxygen vacancies than WP3. This not only increases the intrinsic carrier concentration, but also introduces more active defect sites at and near the surface, thereby markedly enhancing charge transfer and interfacial reactions between adsorbed oxygen species and H_2_, which amplifies the resistance change. However, when the PS content in the starting powder is further increased to 10% and 20%, the O/W ratio decreases further to 2.65 and 2.58, respectively, suggesting that the system becomes excessively reduced. Under this condition, the baseline conductivity is altered and the depletion layer becomes too thin, so the overall resistance can no longer be effectively modulated by surface reactions, leading to a decline in response magnitude. On the other hand, the SEM results show that WP5 develops a uniform and interconnected porous structure while still maintaining a continuous film and good apparent film–substrate adhesion, as suggested by the absence of obvious delamination in cross-sectional SEM images. Nevertheless, quantitative adhesion evaluation, such as scratch testing or tape testing, was not performed in this study and should be included in future device-level reliability assessments. This microstructure not only increases the specific surface area and effective reaction volume, but also shortens the diffusion path of hydrogen within the film, which is beneficial for achieving both high response and rapid kinetics at moderate temperatures. By contrast, in WP10, the rapid release of large amounts of gas during PS pyrolysis gives rise to horizontal cracks and structurally nonuniform regions in the WO_3_ layer. As a result, the current tends to bypass the sensing-active regions through highly reduced domains or crack-related pathways, thereby weakening the control of surface chemical processes over the total resistance. Combined with the temperature-dependent results, WP5 reaches its maximum response at around 120 °C while still exhibiting relatively fast response and recovery, indicating that its oxygen-vacancy concentration, pore architecture, and carrier-transport properties are well matched to the adsorption–reaction–desorption kinetics at this operating temperature. In contrast, WP3 suffers from insufficient reaction activity at the same temperature, whereas in WP10 and WP20 the response amplification effect is partially offset by excessive reduction and structural damage. The superior response of WP5 should be understood as an optimized balance rather than a monotonic consequence of increasing PS content. Moderate PS addition increases the oxygen-vacancy concentration and creates gas-accessible porous structures, thereby enhancing hydrogen activation and charge transfer. However, excessive PS loading leads to over-reduction, stronger local lattice disorder, crack formation, and possible disruption of continuous conduction pathways. These adverse effects offset the benefits of increased porosity and defect density in WP10 and WP20. Therefore, WP5 can be regarded as lying within an optimal window in which the defect population is sufficiently active but not oversaturated, and the porous structure is well developed without compromising film continuity, ultimately giving rise to the best hydrogen-sensing performance in this work. Because PS addition also changes the film thickness, the WP5 optimum may include a thickness-related contribution. A future fixed-thickness sample series will be required to quantitatively separate thickness, porosity, and defect effects. It should be noted that the variation in PS content also changes the final film thickness, which may influence the baseline resistance, gas-diffusion length, and effective reaction volume. Therefore, the sensing performance trend cannot be assigned solely to porosity or defect concentration.

### 3.3. Feasibility Assessment for LIB Safety Monitoring

**Figure 4:**
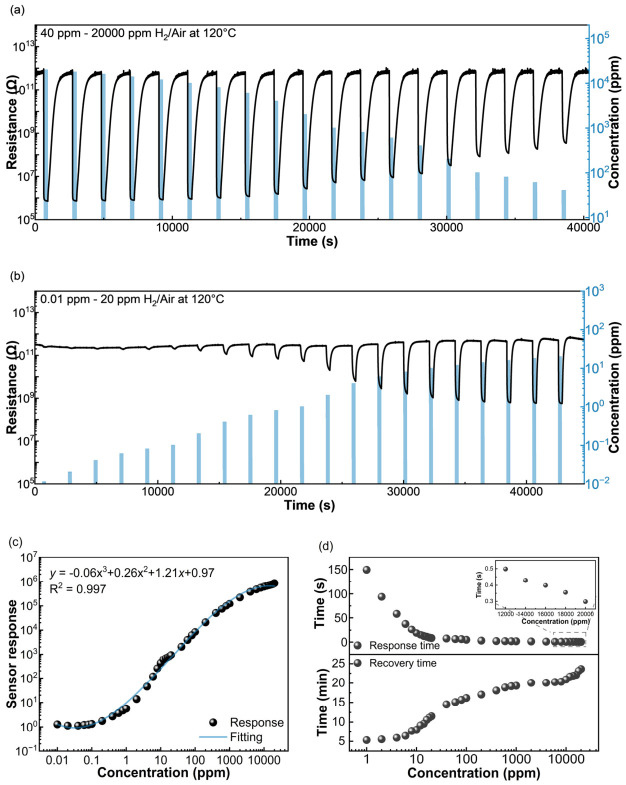
Dynamic response–recovery curves of WP5. (**a**) 40–20,000 ppm; (**b**) 0.01–10 ppm; (**c**) response values and nonlinear fitting results; (**d**) response and recovery time.

After identifying WP5 as the composition with the best overall performance in this system, its dynamic resistance response/recovery behavior toward different hydrogen concentrations was further systematically investigated at the optimal operating temperature of 120 °C. The H_2_ concentration was gradually increased from the ppb level to 2 vol% (20,000 ppm), and the results are shown in Figure 4a,b.

The device exhibited clearly distinguishable resistance transitions during each hydrogen exposure and purge cycle. As the hydrogen concentration increased, the resistance change rose monotonically, and a stable and reproducible response signal could already be obtained at 0.01 ppm (10 ppb), indicating that the WP5 sensor possesses effective ppb-level detection capability and a wide operating concentration range spanning from trace levels to high-concentration shock conditions. Even slight increases in concentration were able to induce stable and reproducible resistance variations, demonstrating that the sensor maintained high concentration resolution and a favorable signal-to-noise ratio even in the ultralow-concentration region. Throughout the entire stepwise test, the response curves remained smooth and continuous, with no obvious noise amplification or baseline drift. Moreover, after completion of the full concentration cycle, the resistance largely returned to its initial value, confirming the good dynamic stability and reversibility of WP5 under continuously varying concentration conditions. More importantly, the observation of reproducible and clearly identifiable stepwise responses even at the ppb level experimentally verifies that the device achieves a detection limit below the ppm range, thereby providing a reliable basis for the subsequent quantitative estimation of the limit of detection from the calibration curve. These results further confirm that the PS-sacrificial-phase-induced bulk reduction and pore-structure optimization significantly enhance the low-concentration hydrogen-sensing capability.

The steady-state response values extracted at different hydrogen concentrations are summarized in Figure 4c,d. It can be seen that the response–concentration relationship exhibits typical nonlinear behavior. An empirical power-law model provides an excellent fit to the experimental data, with a coefficient of determination of R^2^ ≈ 0.997, indicating that the response of the WP5 sample is highly regular and well describable over a wide concentration range, which is advantageous for subsequent quantitative concentration readout through calibration. The response and recovery times at different hydrogen concentrations were further extracted, as shown in Figure 4d. The response time gradually decreases with increasing concentration, which can be attributed to the fact that, under identical gas-flow and delivery conditions, a higher hydrogen partial pressure corresponds to a larger molecular flux, enabling surface active sites to be occupied more rapidly and charge-transfer reactions to proceed more quickly, thereby establishing a new adsorption/reaction dynamic equilibrium in a shorter time. In the low-concentration region, by contrast, the lower collision frequency of hydrogen molecules leads to a longer accumulation time to achieve a comparable modulation of the depletion layer, while the recovery process is simultaneously limited by the slower desorption kinetics and the re-adsorption of background oxygen.

Overall, owing to the synergistic regulation of bulk reduction and porous-structure engineering induced by the PS sacrificial phase, the WP5 sample exhibits not only a high response magnitude and favorable response/recovery kinetics at a moderate operating temperature, but also a well-fit response–concentration relationship and clear dynamic features over a wide concentration range from low ppm levels to percent-level volume fractions. These characteristics provide a solid basis for subsequent evaluation of its selectivity and long-term stability, as well as for its engineering application in the early warning of lithium-ion battery thermal runaway.

**Figure 5:**
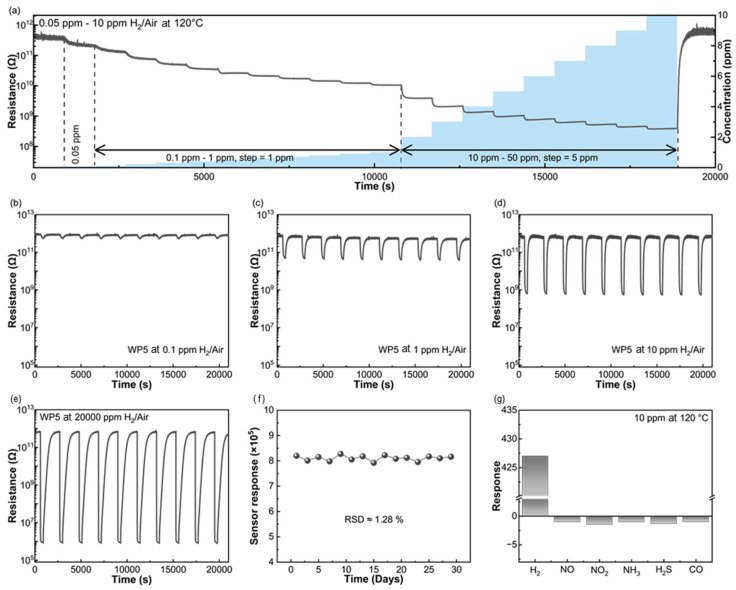
Applicability of WP5. (**a**) Response towards 1–50 ppm with step increase; repeatability under (**b**) 0.1 ppm; (**c**) 1 ppm; (**d**) 10 ppm; and (**e**) 2 vol%. (**f**) Long-term stability and (**g**) selectivity.

To quantitatively evaluate the concentration-discrimination capability and near-linear response behavior of the WP5 device in the ultralow-concentration regime, stepwise H_2_ sensing measurements were conducted at 120 °C over the ppb-to-ppm range, as shown in Figure 5a. During the test, while maintaining a constant operating temperature and total gas flow, the hydrogen concentration was increased stepwise from the ppb level to 10 ppm. In the figure, the step profile represents the programmed variation in the target-gas concentration with time, whereas the solid line corresponds to the real-time resistance response of the sensor. As the ambient H_2_ concentration gradually increased, the sensor resistance decreased monotonically throughout the measurement and exhibited relatively stable resistance plateaus at each target concentration, corresponding to the steady-state response levels. The resistance differences between adjacent steps were clearly distinguishable; even in the ppb regime, slight increases in concentration produced readily resolvable resistance changes, indicating excellent concentration resolution and a high signal-to-noise ratio in the ultralow-concentration range. As the concentration entered the ppm regime, the resistance drop became more pronounced while maintaining a well-defined stepwise pattern, demonstrating that WP5 can effectively resolve and stably track hydrogen variations over a broad concentration window under continuously increasing concentration conditions without full recovery to the baseline between steps. Overall, these step-response results dynamically confirm that PS-sacrificial-phase-induced bulk reduction and porous-structure optimization significantly enhance the low-concentration hydrogen-sensing capability.

To systematically evaluate the repeatability of the Pd/PS–WO_3-x_ sensor at different concentrations, multi-concentration, multi-cycle dynamic H_2_ response/recovery tests were carried out at the optimal operating temperature of 120 °C. Specifically, four representative concentrations, namely 0.1 ppm, 1 ppm, 10 ppm, and 20,000 ppm, were sequentially selected, and ten on–off cycles were performed at each concentration under identical test conditions. During these measurements, the sensor was repeatedly switched between the corresponding hydrogen atmosphere and air, and the representative dynamic response curves are shown in Figure 5b–e. It can be seen that, over the wide concentration range from the ultralow level of 0.1 ppm to the high concentration of 20,000 ppm, both the response magnitude and the response/recovery processes are highly reproducible in each cycle. The response values fluctuate only within a narrow range from cycle to cycle, and no obvious response attenuation, baseline drift, or significant slowing of the response/recovery kinetics is observed with increasing cycle number. In particular, in the low-concentration regime of 0.1, 1, and 10 ppm, each exposure generates a clearly distinguishable and highly consistent resistance-transient profile, indicating that the device maintains excellent signal stability and concentration repeatability even at trace levels. At the high concentration of 20,000 ppm, the response magnitude and recovery level remain essentially unchanged after repeated cycling, suggesting that neither the WO_3_ film nor the Pd catalytic layer undergo noticeable degradation during repeated hydrogen adsorption/desorption processes. Overall, the Pd/PS–WO_3-x_ sensor exhibits outstanding cycling stability and repeatability across a broad concentration range from sub-ppm levels to tens of thousands of ppm, providing strong support for its long-term reliable operation in the early warning of lithium-ion battery thermal runaway and in high-concentration hydrogen leak monitoring. Furthermore, to evaluate the durability of the Pd/PS–WO_3-x_ sensor, a 30-day stability test was performed at 120 °C toward 2 vol% H_2_, and the results are shown in Figure 5f. The response remained nearly constant at around 8.1 × 10^5^ throughout the test, with an RSD of about 1.28%, and no obvious response decay, baseline drift, or kinetic deterioration was observed. These results confirm the excellent long-term reliability of the sensor for practical hydrogen monitoring. The 30-day test demonstrates the good laboratory operating stability of the WP5 sensor under a constant working temperature of 120 °C. However, this test does not fully reproduce practical battery-system conditions, where the sensor may experience thermal cycling, vibration, humidity, and complex vent-gas exposure. Further work will focus on device-level reliability tests, including cyclic heating/cooling, vibration resistance, electrical-contact stability, and operation under realistic battery vent-gas environments.

Selectivity is a key criterion for evaluating the ability of a gas sensor to distinguish the target gas from interfering species. To assess the anti-interference performance of WP5 under practical conditions, comparative tests were carried out toward H_2_, CO, H_2_S, NH_3_, NO, and NO_2_ under identical conditions, and the results are shown in Figure 5g. At 120 °C and the same gas concentration, the response to H_2_ exceeds 400, whereas those to CO, H_2_S, NH_3_, NO, and NO_2_ are all below 1.5, more than two orders of magnitude lower than that toward H_2_. In addition, the resistance change induced by these interfering gases is opposite in direction to that caused by H_2_, further highlighting the distinct sensing behavior of the device. This high selectivity can be mainly attributed to the Pd catalytic layer, which promotes the dissociation of H_2_ and the subsequent spillover of atomic hydrogen onto WO_3-x_, thereby strongly modulating the depletion layer and carrier concentration. It is also closely related to the intrinsic hydrogen-sensitive nature of WO_3_, since hydrogen insertion induces coupled changes in conductivity and local structure, whereas other interfering gases cannot trigger similarly pronounced reactions under the same conditions. Moreover, the relatively low operating temperature suppresses side reactions of interfering gases, enabling WP5 to achieve highly selective H_2_ detection suitable for lithium-ion battery safety monitoring. Long-term stability is another important indicator for practical applications.

### 3.4. Sensing Mechanism

**Figure 6:**
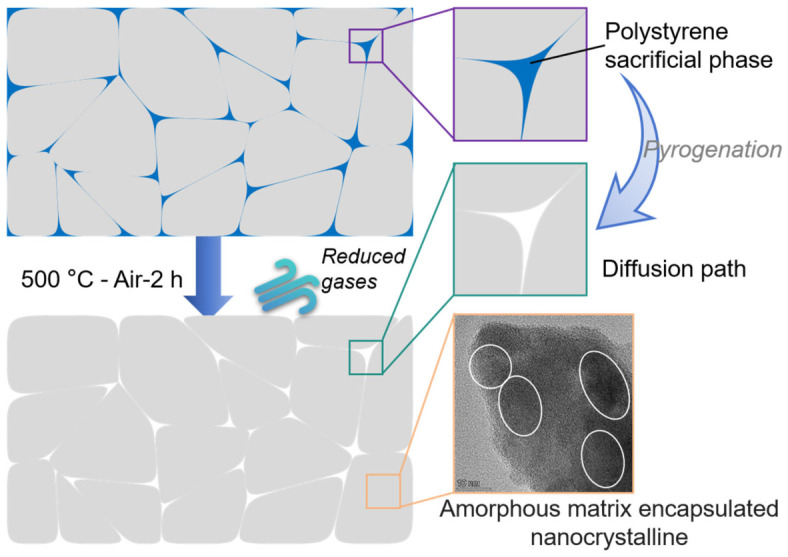
Schematic diagram of PS sacrificial phase introduction mechanism.

From the perspective of the hydrogen-sensing mechanism, the results of this work indicate that the introduction of the PS sacrificial phase provides two key structural advantages to the WO_3_ films, which further act synergistically with the amorphous matrix encapsulated nanocrystalline structure formed by PAD, ultimately leading to enhanced sensing performance, as illustrated in Figure 6. First, PS is co-deposited as a space-occupying phase during deposition and is completely removed after sintering in air, thereby generating interconnected pores and a rough porous interface throughout the film interior and surface. This structure markedly increases the effective reaction area and shortens the diffusion path of hydrogen within the film, resulting in an enhanced response magnitude and accelerated response/recovery kinetics [28]. Second, the reducing species released during PS pyrolysis create a localized reducing atmosphere inside the film, promoting bulk oxygen loss in WO_3_ and increasing the concentrations of oxygen vacancies and W^5+^ species. As a result, the material evolves from a near-stoichiometric state toward WO_3-x_, which enhances the activation of adsorbed oxygen species and strengthens hydrogen-induced charge transfer, while also shifting the optimum operating temperature to a lower range. In addition to creating voids, the removal of PS may locally modify the WO_3_ structure near the pore walls. Although XRD confirms that the main γ-WO_3_ phase is retained, the weakening of the low-frequency monoclinic Raman mode and the broadening of W–O vibrational bands indicate increased local disorder and WO_6_-octahedra distortion with increasing PS content. Therefore, the PS-derived pores are likely surrounded by oxygen-deficient and locally distorted WO_3-x_ regions, which can provide additional active sites for hydrogen interaction. However, excessive PS loading may induce over-reduction and structural damage, explaining the degraded performance of highly PS-loaded samples.

Notably, the sintering temperature and holding time adopted in this work are relatively mild. Thus, while enabling PS removal and defect regulation, they do not significantly disrupt the PAD-derived microstructure composed of nanocrystals embedded in an amorphous matrix. This preserved composite framework maintains continuous charge-transport pathways and structural integrity, while simultaneously benefiting from the dual effects of porous diffusion channels and bulk defect activation. Overall, the pore-structure construction and bulk oxygen-vacancy modulation induced by the PS sacrificial phase, together with the retention of the PAD amorphous matrix encapsulated nanocrystalline structure, are the main reasons why the samples in this work exhibit higher response, faster kinetics, and a lower optimum operating temperature compared to other hydrogen sensors, as shown in Table 1. It should be noted that the present measurements were conducted using certified laboratory gas mixtures under controlled humidity-free conditions. Real lithium-ion battery vent gas contains multiple components, such as CO, CO_2_, CH_4_, C_2_H_4_, electrolyte vapors, aerosols, and water vapor. Therefore, the present results demonstrate the material-level feasibility of Pd/PS–WO_3-x_ sensors for early H_2_ warning, while further validation under real battery vent-gas conditions is required before practical deployment.

## 4. Conclusions

Following sacrificial-template regulation and bulk reduction as the central theme, this work introduces a polystyrene organic sacrificial phase into the PAD process to construct PS–WO_3_ composite precursor powders, and realizes the synergistic tuning of pore architecture and oxygen stoichiometry in WO_3_ films through one-step PAD followed by annealing in air. Structural and chemical analyses by XRD, Raman, SEM, and XPS show that the PS phase can be completely removed during sintering without altering the main γ-WO_3_ crystal phase, while simultaneously generating interconnected porous channels and inducing controllable bulk reduction from near-stoichiometric WO_3_ toward WO_3-x_. Importantly, the PAD-derived amorphous matrix encapsulated nanocrystalline framework is largely retained, providing a stable structural basis for gas sensing. As a result, pore formation, oxygen-vacancy enrichment, and preservation of the PAD composite skeleton act together to improve H_2_ transport, surface reaction activity, and charge-transfer efficiency.

Among all compositions, the optimized WP5-based Pd/PS–WO_3-x_ sensor exhibits stable H_2_ detection over a broad concentration range from 10 ppb to 20,000 ppm at a moderate operating temperature of 120 °C, together with high response magnitude, rapid response/recovery behavior, excellent repeatability, strong selectivity against common interfering gases, and good laboratory stability over 30 days. These results demonstrate that PS-assisted microstructure and defect engineering is an effective strategy for improving the hydrogen-sensing performance of PAD-derived WO_3-x_ films. In addition, the device shows excellent selectivity against common interfering gases, high repeatability over multiple concentration cycles, and good long-term stability over 30 days with an RSD of about 1.28%. These results demonstrate that the PS-sacrificial-template strategy is an effective route for simultaneously engineering porosity and defect chemistry in WO_3_-based sensing films, and further confirms the strong potential of PAD-derived Pd/PS–WO_3-x_ sensors for practical hydrogen monitoring and early warning of lithium-ion battery thermal runaway. To further advance this material platform toward practical lithium-ion battery safety monitoring, future work should focus on sensor miniaturization, integration with battery-management systems, validation in real battery vent-gas atmospheres, evaluation under humidity and electrolyte-vapor interference, and reliability testing under thermal cycling, vibration, and packaging-induced mechanical stress.

## Figures and Tables

**Table 1:** Performance comparison of various hydrogen sensing materials.

Material	Range (ppm)	S	NS (/ppm)	T90(s)	WT (°C)	Ref
Pd/WO_3_	0.01–20,000	8.1 × 10^5^ at 20,000 ppm	~40.5	0.3	120	This Work
Pd/WO_3_-SnO_2_	0.5–100	235.52 at 50 ppm	~4.7	1	90	[29]
Pd-Na/WO_3_	0.2–100	218 at 25 ppm	~8.7	3	110	[30]
Pd-WO_3_/WS_2_	200–1000	4227.35 at 1000 ppm	~4.2	1	125	[31]
Pd-WO_3_	20–1000	8658.98 at 500 ppm	~17.3	1	150	[32]

NS: Normalized response; WT: Working temperature.

## Data Availability

The original contributions presented in this study are included in the article/Supplementary Materials. Further inquiries can be directed to the corresponding authors.

## Supplementary Materials

The following supporting information can be downloaded at: https://www.mdpi.com/article/10.3390/ma19143079/s1, Table S1. Experimental parameter of the PAD process in this chapter; Table S2; The volume ratio of PS mixed WO_3_ starting powder; Figure S1. Schematic illustration of sample preparation; Figure S2. Structural characterization of PS starting powder (a) XRD; (b) Raman; Figure S3. SEM images of PS starting powder. (a) 5 k×; (b) 20 k×; Figure S4. XPS spectrum of different WO3 thin films; Figure S5. The dynamic sensing performance of all samples under 2% vol H_2_/Air (a) 40 °C; (b) 60 °C; (c) 80 °C; (d) 100 °C; (e) 120 °C; (f) 140 °C; (g) 160 °C; (h) 180 °C; (i) 200 °C.
