# Supplementary material for: Polystyrene-Templated Microstructure Engineering of Aerosol-Deposited WO3-x Films for Enhanced Hydrogen Sensing
Source: Materials (Basel). 2026 Jul 17;19(14):3079. doi: 10.3390/ma19143079 (PMC13413647; doi:10.3390/ma19143079)
Supplement: Supplementary file 1 [file materials-19-03079-s001.zip › materials-4406137-supplementary.pdf]

Polystyrene-Templated Microstructure Engineering of Aerosol-Deposited WO_{3-x} Films for Enhanced Hydrogen Sensing

Xin Zhang [1,†], Yuan-Bo Zhang [2,†], Jong-Min Oh [2,*] and Jie Wei [3,4,*]

[1] Suzhou Institute of Biomedical Engineering and Technology, Chinese Academy of Sciences, Suzhou 215163, China; zhangx@sibet.ac.cn
[2] Department of Electronic Materials Engineering, Kwangwoon University, Seoul 01897, Republic of Korea
[3] School of Electronic and Information Engineering, Suzhou Polytechnic University, Suzhou 215104, China
[4] Suzhou Sanse Sensing Technology Co., Ltd., Suzhou 215000, China
* Correspondence: jmoh@kw.ac.kr (J.-M.O.); weijie9685@foxmail.com (J.W.)
† These authors contributed equally to this work.

Key words: Aerosol deposition; hydrogen sensor; WO_{3-x}; polystyrene sacrificial phase

Table S1. Experimental parameter of the PAD process in this chapter

Parameters	Conditions
Starting powder	PS-WO_3
Substrate	Quartz glass（6×12 mm）
Injected gas consumption	3 L/min
Type of injected gas	He
Working pressure	2.7 Torr
Deposition temperature	RT
Vibration	400–500 rpm
Scanning number	2 scans
Scanning time	10 min

Table S2. The volume ratio of PS mixed WO_3 starting powder

Name	Volume fraction of PS	Mass fraction of PS
WP3	3%	≈ 0.458 wt%
WP5	5%	≈ 0.762%
WP10	10%	≈ 1.595%
WP20	20%	≈ 3.518%

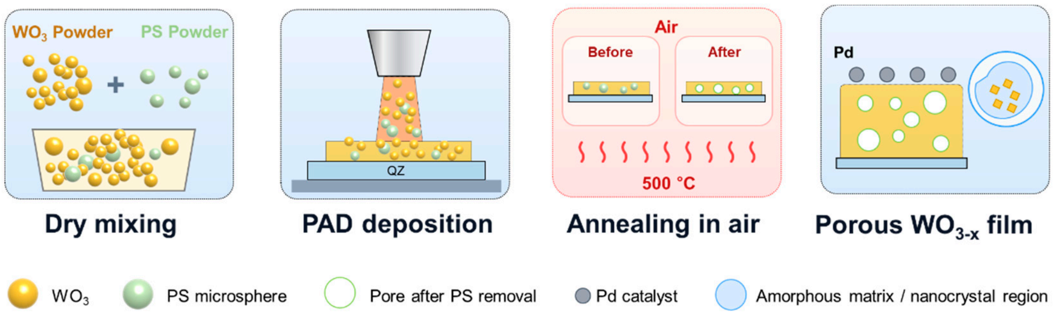

Figure S1. Schematic illustration of sample preparation

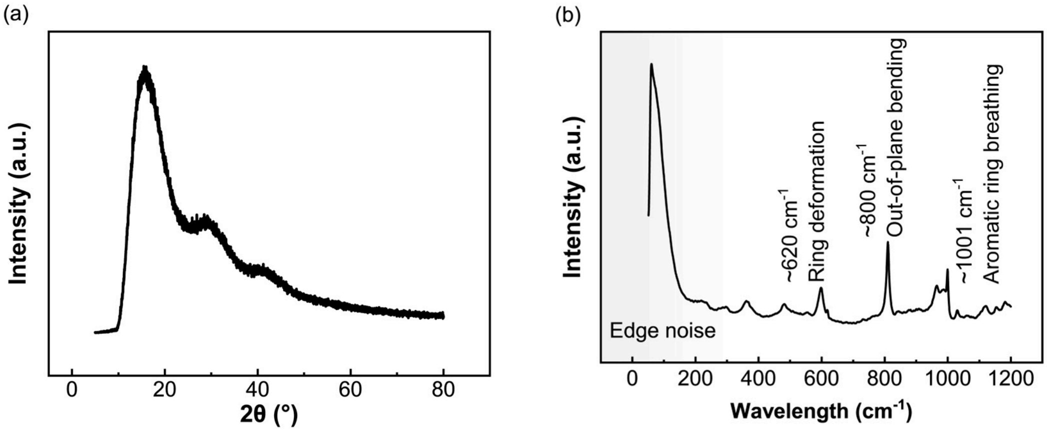

Figure S2. Structural characterization of PS starting powder

(a) XRD; (b) Raman

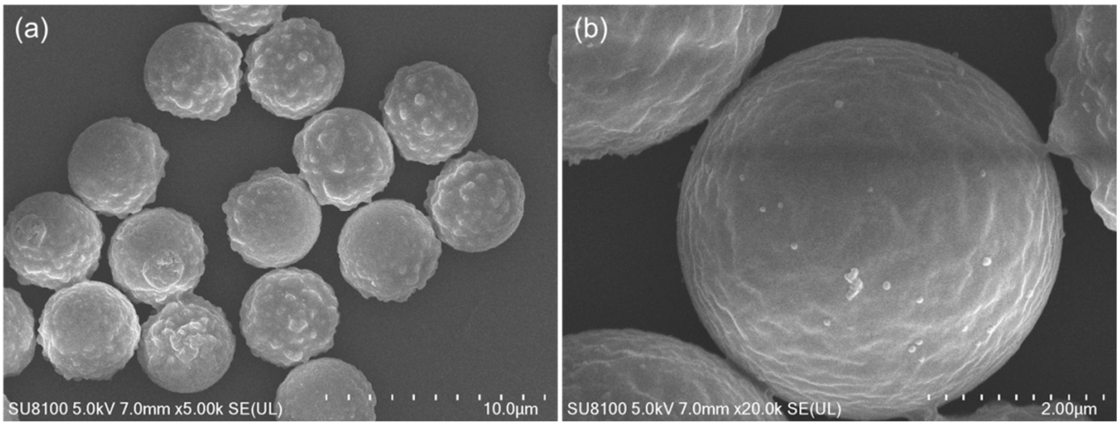

Figure S3. SEM images of PS starting powder. (a) 5k×; (b) 20k×.

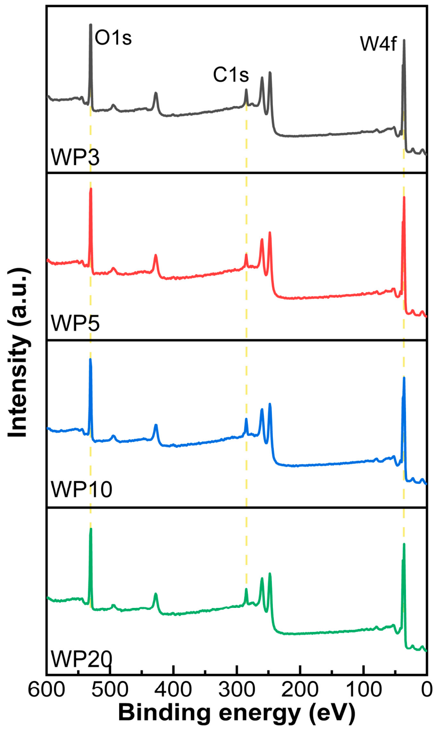

Figure S4. XPS spectrum of different WO_3 thin films

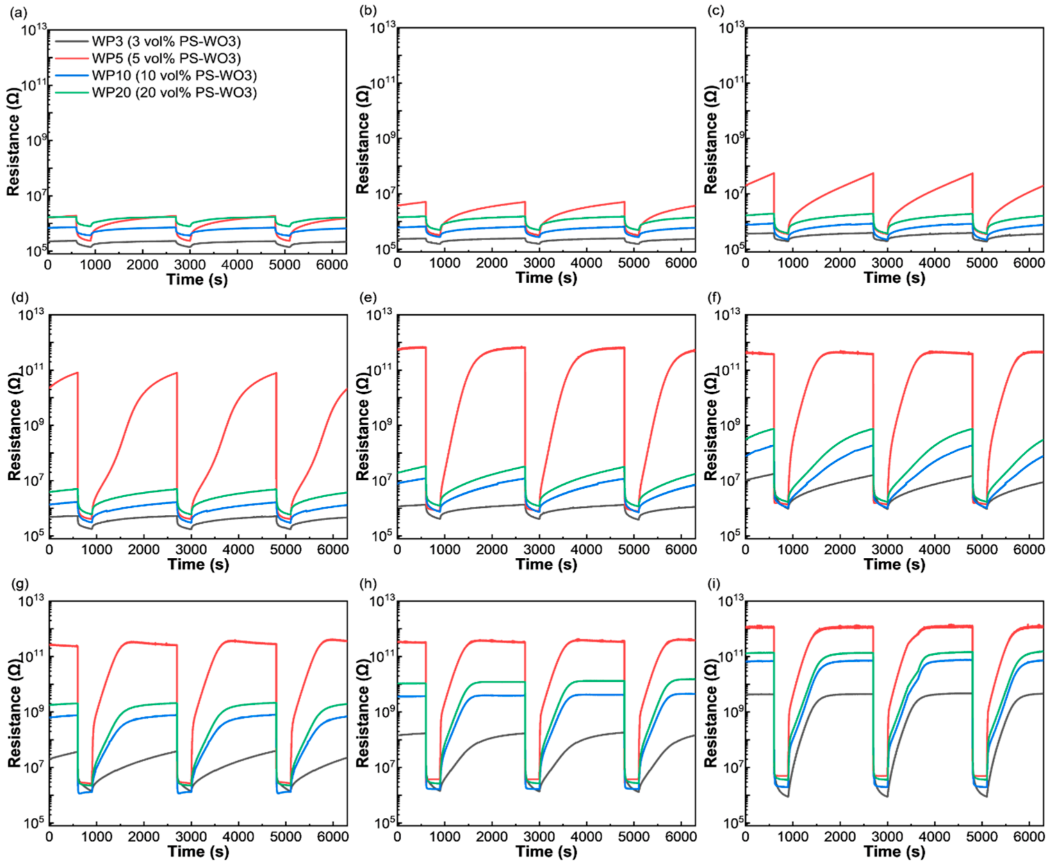

Figure S5 The dynamic sensing performance of all samples under 2% vol H_2/Air

(a) 40 °C; (b)60 °C; (c) 80 °C; (d) 100 °C; (e) 120 °C; (f) 140 °C; (g) 160 °C; (h) 180 °C; (i) 200 °C
